# A viable hypomorphic mutation in the mitochondrial ribosome subunit, MRPS-31, exhibits mitochondrial dysfunction in *C. elegans*

**DOI:** 10.17912/micropub.biology.001344

**Published:** 2024-09-30

**Authors:** Kylie M. Jozwik, James P. Held, Chloe A. Hecht, Maulik R. Patel

**Affiliations:** 1 Department of Biological Sciences, Vanderbilt University, Nashville, Tennessee, United States; 2 Department of Cell and Developmental Biology, Vanderbilt University, Nashville, Tennessee, United States; 3 Evolutionary Studies, Vanderbilt University, Nashville, Tennessee, United States; 4 Diabetes Research and Training Center, Vanderbilt University Medical Center, Nashville, Tennessee, United States

## Abstract

The mitochondrial ribosome (mitoribosome) translates mitochondrial genome encoded proteins essential for cellular energy production. Given this critical role, defects in the mitoribosome can cause mitochondrial stress and manifest as multisystemic diseases. In a screen for unique activators of the mitochondrial unfolded protein response (UPR ^mt^ ) in *Caenorhabditis elegans* , we recovered a strain harboring a missense mutation in the gene encoding mitochondrial ribosome protein S31 ( MRPS-31 )—a component of the mitoribosome small subunit. Herein, we confirm causality of the *mrps-31* allele and characterize its induction of UPR ^mt^ and impact on organismal development, providing a valuable model for further study of the mitoribosome.

**Figure 1.**
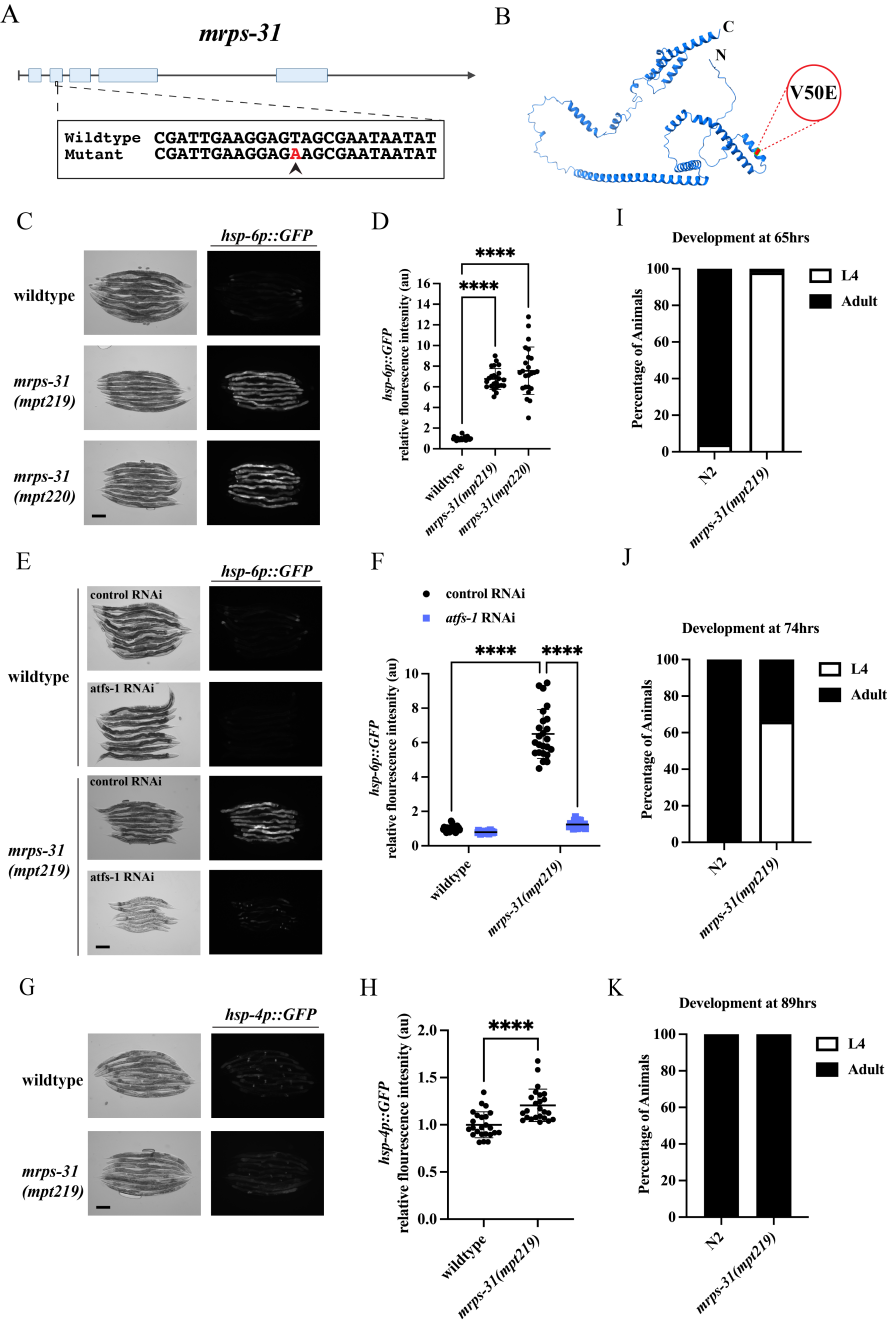
(A) Schematic of the *C. elegans* *mrps-31* gene structure, highlighting the T>A mutation responsible for UPR ^mt^ reporter activation in strains harboring the *mpt219* and *mpt220* alleles. (B) AlphaFold predicted structure of the *C. elegans* MRPS-31 from UniProt (UniProt Consortium, 2023), with the V50E mutation site marked in red. (C) Corresponding brightfield and fluorescence images of UPR ^mt^ reporter ( *hsp-6 p::GFP* ) activation in day 2 adult (D2A) wildtype, *mrps-31* ( *mpt219* ), and *mrps-31* ( *mpt220* ) animals. Scale bar 200 μm. (D) Fluorescence intensity quantification of *hsp-6 p::GFP* reporter activation in individual D2A wildtype, *mrps-31* ( *mpt219* ), and *mrps-31* ( *mpt220* ) animals normalized to *hsp-6 p::GFP* in a wildtype background (n=24 for each condition, au=arbitrary units, mean and SD shown, ordinary one-way ANOVA with Tukey's multiple comparison test). (E) Corresponding brightfield and fluorescence images of UPR ^mt^ reporter ( *hsp-6 p::GFP* ) activation in D2A wildtype and *mrps-31* ( *mpt219* ) animals on control and *atfs-1* RNAi. Scale bar 200 μm. (F) Fluorescence intensity quantification of *hsp-6 p::GFP* in individual D2A wildtype and *mrps-31* ( *mpt219* ) animals on control and *atfs-1* RNAi normalized to *hsp-6 p::GFP* in a wildtype background on control RNAi (n=24 for each condition, au=arbitrary units, mean and SD shown, two-way ANOVA with Tukey's multiple comparisons test). (G) Corresponding brightfield and fluorescence images of UPR ^ER^ reporter ( *hsp-4p::GFP* ) activation in day 2 adult (D2A) wildtype and *mrps-31* ( *mpt219* ) animals. Scale bar 200 μm. (H) Fluorescence intensity quantification of *hsp-4p::GFP* reporter activation in individual D2A wildtype and *mrps-31* ( *mpt219* ) animals normalized to *hsp-4p::GFP* in a wildtype background (n=24 for each condition, au=arbitrary units, mean and SD shown, unpaired t-test). (I-K) Percentage of N2 and *mrps-31 ( mpt219 )* animals that have reached L4/adulthood at 65hrs, 74hrs, and 89hrs respectively (n=50).

## Description

Over 1,100 proteins are necessary to make a functional mitochondrion (Rath et al., 2021) . Of these, 99% are encoded by the nucleus and thereafter targeted to the mitochondria while the remaining few are encoded by the mitochondrial genome (mtDNA) (Weinhouse, 2017) . The 13 proteins encoded by mammalian mtDNA are critical components of the electron transport chain, necessary for creating the electrochemical gradient that results in the production of ATP—the major source of cellular energy. Unlike nuclear-encoded mitochondrial proteins, the translation of mtDNA encoded proteins depends on the mitochondrial ribosome (mitoribosome), a distinct and specialized ribosome unique from its cytosolic counterpart (Greber & Ban, 2016) .

Given the role of the mitoribosme in translating mtDNA-encoded proteins, dysregulation of any of the 82 nuclear-encoded mitoribosomal proteins (MRPs) can lead to significant mitochondrial stress (De Silva et al., 2015) . In fact, mutations in MRPs can manifest as multisystemic human diseases including sensorineural hearing loss, hypertrophic cardiomyopathy, and neurological deterioration, due to disrupted mitochondrial bioenergetics (Rötig, 2011; De Silva et al., 2015) . Therefore, continued study and characterization of MRPs remain important for understanding basic mitochondrial biology and associated human health.

In response to mitochondrial dysregulation, cells activate retrograde signaling pathways to mitigate stress (Ng et al., 2021) . One such mechanism is the conserved mitochondrial unfolded protein response (UPR ^mt^ ). UPR ^mt^ promotes survival and recovery of the mitochondrial network by activating signaling cascades that result in the upregulation of nuclear-transcribed chaperones and proteases (Shpilka & Haynes, 2018) . In *Caenorhabditis elegans* , where UPR ^mt^ is well characterized, UPR ^mt^ activationis dependent on the dual-targeted transcription factor, ATFS-1 . In healthy cells, ATFS-1 is predominantly imported into the mitochondria (Nargund et al., 2012) . However, under conditions of mitochondrial stress, ATFS-1 traffics to the nucleus where it drives the expression of mitochondrial-protective genes (Nargund et al., 2015) . This mechanism has been leveraged in *C. elegans* to develop a transgenic reporter strain in which one of the targets of ATFS-1 , the *hsp-6* promoter, drives the expression of GFP (Yoneda et al., 2004) . Therefore, GFP expression in this strain is a proxy for UPR ^mt^ activation.

Previously, we utilized the *hsp-6 p::GFP* reporter to conduct a forward genetic screen for mutations that activate the mitochondrial unfolded protein response (UPR ^mt^ ) specifically in the intestine of *C. elegans* (Held et al., 2024) *.* Six independent mutants were recovered, one of which ( MRP650 ), harbored a putative causal missense mutation in mitochondrial ribosome protein S31 ( *mrps-31 ),* homolog of the human *MRPS31,* which encodes a component of the small subunit of the mitoribosome. However, as is typical with random mutagenesis, there are other mutations linked to the *mrps-31* variant. To determine if the MRPS-31 V50E mutation found in MRP650 was causal in activating UPR ^mt^ ( Figure 1A, 1B), we introduced it into a N2 wildtype genetic background using CRISPR/Cas9 and assessed *hsp-6 p::GFP* reporter activation in live animals. We recovered and sequence verified two independent, MRPS-31 V50E CRISPR hits (alleles *mpt219* and *mpt220* , denoted *mrps-31* ( *mpt219* ) and *mrps-31* ( *mpt220* )), and fluorescence microscopy revealed similarly robust UPR ^mt^ reporter activation in adult animals of both alleles ( Figure 1C, 1D). Thus, we further characterized one of these alleles: *mrps-31* ( *mpt219* ). Knockdown of ATFS-1 , the central transcription factor of UPR ^mt^ , completely abrogates UPR ^mt^ activation in *mrps-31* ( *mpt219* ) animals ( Figure 1E, 1F). In addition, *mrps-31* ( *mpt219* ) animals on *atfs-1* RNAi are smaller and display developmental defects. These data suggest that MRPS-31 V50E is the causal mutation in strain MRP650 recovered from the screen and that it activates canonical ATFS-1-dependent UPR ^mt^ .

To determine whether the stress induced by *mrps-31* ( *mpt219* ) is specific to the mitochondria, we also assessed activation of the endoplasmic reticulum unfolded protein response (UPR ^ER^ ) reporter, *hsp-4p::GFP* —a proxy for ER stress and compromised cellular proteostasis (Calfon et al., 2002) . We find that *hsp-4p::GFP* reporter is only very slightly induced in *mrps-31* ( *mpt219* ) animals, much less than what is observed by known activators of UPR ^ER^ ( Figure 1G, 1H) (Calfon et al., 2002; Hou et al., 2014) . These data suggest that stress induced by *mrps-31* ( *mpt219* ) is predominantly specific to the mitochondria.

Mitochondrial stress often correlates with delayed organismal development (Feng et al., 2001; Yang & Hekimi, 2010) . Thus, we assessed development of *mrps-31* ( *mpt219* ) animals. The N2 wildtype *C. elegans* strain develop from a fertilized egg into a reproductively mature, egg laying hermaphrodite adults in approximately 65hrs at 20°C (Byerly et al., 1976) . Therefore, developmental stage was assessed at 65hrs at which time 96% of N2 animals were reproductive adults while only 2% of *mrps-31* ( *mpt219* ) animals had reached adulthood ( Figure 1I ). Developmental stage was also assessed at 74hrs where 34% of *mrps-31* ( *mpt219* ) animals reached reproductive maturity and at 89hrs, where all animals had reached reproductive adulthood ( Figure 1J, 1K).

It is known that compromising mitoribosome function leads to mitochondrial stress and dysfunction (Rolland et al., 2019; Lopez Sanchez et al., 2021) . However, mitoribosome defects can be challenging to study because these genes are essential thus rendering homozygous mutations non-viable. For example, the National BioResource Project isolated a *C. elegans* strain with a 450bp deletion in exon 1 of *mrps-31* (allele: *tm1314* ) but these animals have a sterile/lethal phenotype (Mitani, 2009) . Herein, we have identified and characterized a hypomorphic *mrps-31* allele in *C. elegans ,* which induces mitochondrial stress yet is reproductively viable and able to be maintained as a homozygous population. UPR ^mt^ reporter activation and slowed development phenotype of *mrps-31* ( *mpt219* ) animals is consistent with a loss-of-function mutation. Interestingly, *mrps-31* ( *mpt219* ) exhibits robust UPR ^mt^ reporter activation predominantly in the intestine of *C. elegans* , which is unique compared to other systemic mutations that impair electron transport chain function which activate UPR ^mt^ in all somatic tissues (Baker et al., 2012; Held et al., 2024) . This tissue specific activation suggests that MRPS-31 may not be required equally across all tissues or that *mrps-31* ( *mpt219* ) selectively impairs function in the intestine. Our recovery of a mutation in a mitoribosome subunit that exhibits features of mitochondrial stress yet is reproductively viable provides a valuable system for studying mitoribosome biology and has potential to serve as a model for disorders associated with mitoribosome dysfunction.

## Methods

Worm Maintenance- Animals were grown on nematode growth media (NGM) plates seeded with OP50 *E.coli* obtained from the Caenorhabditis Genetics Center. All strains were maintained at 20°C.

CRISPR/Cas9- CRISPR was conducted using Alt-R S.p. Cas9 Nuclease V3 (IDT#1081058) and tracrRNA (IDT#1072532) as previously described (Dokshin et al., 2018) . Instead of using *rol-6* plasmid, we used *dpy-10* endogenous editing as a co-injection marker as previously described (Paix et al., 2015) . Once the desired edit was recovered, the *dpy-10* injection marker was outcrossed using a wildtype background ( N2 ).

Genetic Crosses - Strains resulting from genetic crosses were generated by crossing 15-20 heterozygous males of a given strain to 5-8 larval stage 4 (L4) hermaphrodites of another strain (heterozygous males were first generated by crossing wildtype N2 males to L4 hermaphrodites of a strain). F1 generation L4s were cloned out from the cross plates. Once F2 progeny were laid, the F1 adult was genotyped and screened for the alleles of interest. F2 progeny were cloned out from F1 plates harboring the allele(s) of interest and once F3 progeny were laid, the F2 animals were genotyped and screened again for the alleles of interest.

RNAi - RNAi by feeding was conducted as previously described (Held et al., 2022) . Briefly, *atfs-1* and empty vector RNAi clones were grown up overnight at 37°C shaking from a single colony in 2mL liquid culture of LB supplemented with 50μg/ml ampicillin. To make 16 RNAi plates, 50mL of LB supplemented with 50μg/ml ampicillin was inoculated with 500μL of overnight culture and then incubated shaking overnight at 37°C. Following overnight incubation, cultures were induced by adding an additional 50mL of LB supplemented with 50μg/ml ampicillin in addition to 8mM IPTG and then incubated shaking at 37°C for 3.5-4hrs (to an OD _550-600_ of approximately .8). After incubation, the OD _550-600_ was taken and the cultures were pelleted by centrifugation at 3900 rpm for 6min. Supernatant was removed and a mixture of 4mL of M9 supplemented with 8mM IPTG was made. Pellets were resuspended in the M9+IPTG mixture such that the final OD was standardized to 0.8. 250μL of suspension was seeded onto standard 60mm NGM plates containing 1mM of IPTG. Plates were left to dry overnight and then used within 1 week. Bacterial RNAi feeder strain was from the Ahringer RNAi Feeding Library, grown from a single colony and identity confirmed by Sanger sequencing.

Fluorescence Microscopy - All animal imaging was performed using a Zeiss Axio Zoom V16 stereo zoom microscope. Worms were immobilized on 2% agarose pads on microscope slides in ~1μL of 100mM levamisole (ThermoFisher #AC187870100) and then a coverslip was applied.

Image Quantification - All fluorescent microscopy images were analyzed with FIJI. First, a region of interest was drawn around the body wall of each worm using the brightfield image. Subsequently, the corresponding fluorescent image was opened in the program and average fluorescent intensity of each individual biological replicate was obtained using the measure function (which calculates the average pixel intensity by dividing the sum total fluorescent intensity by the total number of pixels within the bounds of the trace) for each region of interest.

Development Assay - 25 gravid adult animals were plated on OP50 seeded 60mm NGM plates and allowed to lay embryos for 90min at room temperature. Following egg laying, 50 embryos per genotype were moved to a clean OP50 seeded NGM plate and incubated at 20°C for 65 hrs. After incubation, development of wildtype and mutant animals were assessed. At this time, all animals were L4 stage (characterized by the developing vulva) or later. Adulthood was determined by the presence of a line of embryos flanking the vulva or the presence of vulval eversion (the final stage of vulva morphogenesis).

## Reagents

**Table:** 

Strain	Genotype	Available from
N2	*wildtype*	CGC
MRP650	*mpt140* ; *zcIs13* V	Held et al., 2024
GL347	*zcIs13* [ *hsp-6 p* ::GFP] V	CGC
SJ4005	*zcIs4* [ *hsp-4p* ::GFP] V	CGC
MRP1054	*mrps-31* ( *mpt219* [V50E]) III	This study
MRP1055	*mrps-31* ( *mpt219* [V50E]) III; *zcIs13* V	This study
MRP1057	*mrps-31* ( *mpt220* [V50E]) III; *zcIs13* V	This study
MRP1056	*mrps-31* ( *mpt219* [V50E]) III; *zcIs4* V	This study
